# Phylogenetic Analysis of Invasive Serotype 1 Pneumococcus in South Africa, 1989 to 2013

**DOI:** 10.1128/JCM.00055-16

**Published:** 2016-04-25

**Authors:** Mignon du Plessis, Mushal Allam, Stefano Tempia, Nicole Wolter, Linda de Gouveia, Claire von Mollendorf, Keith A. Jolley, Nontombi Mbelle, Jeannette Wadula, Jennifer E. Cornick, Dean B. Everett, Lesley McGee, Robert F. Breiman, Rebecca A. Gladstone, Stephen D. Bentley, Keith P. Klugman, Anne von Gottberg

**Affiliations:** a Centre for Respiratory Diseases and Meningitis, National Institute for Communicable Diseases, National Health Laboratory Service, Johannesburg, South Africa; b School of Pathology, Faculty of Health Sciences, University of the Witwatersrand, Johannesburg, South Africa; c Influenza Division, Centers for Disease Control and Prevention, Atlanta, Georgia, USA; d Influenza Program, Centers for Disease Control and Prevention, Pretoria, South Africa; e School of Public Health, Faculty of Health Sciences, University of the Witwatersrand, Johannesburg, South Africa; f Department of Zoology, University of Oxford, Oxford, United Kingdom; g Department of Medical Microbiology, Tshwane Academic Hospital, National Health Laboratory Service, Pretoria, South Africa; h Department of Clinical Microbiology and Infectious Diseases, Chris Hani Baragwanath Academic Hospital, National Health Laboratory Service and University of the Witwatersrand, Johannesburg, South Africa; i Institute of Infection and Global Health, University of Liverpool, Liverpool, United Kingdom; j Malawi-Liverpool-Wellcome Trust Clinical Research Programme, Blantyre, Malawi; k Streptococcus Laboratory, Centers for Disease Control and Prevention, Atlanta, Georgia, USA; l Emory Global Health Institute, Emory University, Atlanta, Georgia, USA; m Pathogen Genomics, Wellcome Trust Sanger Institute, Hinxton, United Kingdom; n Hubert Department of Global Health, Rollins School of Public Health, and Division of Infectious Diseases, School of Medicine, Emory University, Atlanta, Georgia, USA

## Abstract

Serotype 1 is an important cause of invasive pneumococcal disease in South Africa and has declined following the introduction of the 13-valent pneumococcal conjugate vaccine in 2011. We genetically characterized 912 invasive serotype 1 isolates from 1989 to 2013. Simpson's diversity index (D) and recombination ratios were calculated. Factors associated with sequence types (STs) were assessed. Clonal complex 217 represented 96% (872/912) of the sampled isolates. Following the introduction of the 13-valent pneumococcal conjugate vaccine (PCV13), ST diversity increased in children <5 years (D, 0.39 to 0.63, *P* = 0.002) and individuals >14 years (D, 0.35 to 0.54, *P* < 0.001): ST-217 declined proportionately in children <5 years (153/203 [75%] versus 21/37 [57%], *P* = 0.027) and individuals >14 years (242/305 [79%] versus 96/148 [65%], *P* = 0.001), whereas ST-9067 increased (4/684 [0.6%] versus 24/228 [11%], *P* < 0.001). Three subclades were identified within ST-217: ST-217_C1_ (353/382 [92%]), ST-217_C2_ (15/382 [4%]), and ST-217_C3_ (14/382 [4%]). ST-217_C2_, ST-217_C3_, and single-locus variant (SLV) ST-8314 (20/912 [2%]) were associated with nonsusceptibility to chloramphenicol, tetracycline, and co-trimoxazole. ST-8314 (20/912 [2%]) was also associated with increased nonsusceptibility to penicillin (*P* < 0.001). ST-217_C3_ and newly reported ST-9067 had higher recombination ratios than those of ST-217_C1_ (4.344 versus 0.091, *P* < 0.001; and 0.086 versus 0.013, *P* < 0.001, respectively). Increases in genetic diversity were noted post-PCV13, and lineages associated with antimicrobial nonsusceptibility were identified.

## INTRODUCTION

Streptococcus pneumoniae serotype 1 is one of the commonest causes of invasive pneumococcal disease (IPD) in Africa, Asia, and South America (1, 2). It usually affects otherwise healthy older children and young adults (3–5) and has been associated with explosive outbreaks of invasive disease (6–8). Clinically, serotype 1 is associated with bacteremia, empyema, and peritonitis and has a lower risk of mortality relative to other serotypes (5, 9–11). It has high invasive potential and is rarely detected in colonization studies, remaining predominantly susceptible to antimicrobial agents (12, 13).

In South Africa, the 13-valent pneumococcal conjugate vaccine (PCV13), which includes serotype 1, replaced PCV7 in mid-2011 (14). A recent epidemiological study in South Africa (5) reported two spatially and temporarily distinct serotype 1 clusters (with rates significantly higher than the expected baseline) in 2003 to 2004 and 2008 to 2012; however, a decrease in serotype 1 incidence was reported in all age groups following PCV13 introduction, even compared to years without clusters.

Using multilocus sequence typing (MLST), four geographically distinct serotype 1 lineages have been described, namely, ST-306 in Europe and North America, ST-227 in the United States, ST-615 in Chile, and ST-217 in Africa and Israel (15, 16). A more recent analysis of 448 serotype 1 genomes from 27 countries (including 58 South African isolates from 2004 to 2008) confirmed the geographic distribution of these lineages and the continued circulation of ST-217 in South Africa (17).

MLST has been the gold standard for molecular typing for almost 2 decades (18). Nevertheless, its power to differentiate lineages is potentially limited by the inclusion of only seven housekeeping loci and thus may not always accurately reflect the relatively recent evolutionary biology of an organism (19). Whole-genome sequencing allows for more in-depth analyses of MLST-defined lineages. Pneumococci are naturally transformable, and genetic variation occurs predominantly by horizontal gene transfer and recombination (20). Antibiotic exposure remains a key selective pressure driving the emergence of recombination-driven resistance (21, 22). In addition, immunological pressure, as a result of pneumococcal immunization, is a theoretical concern for yielding strains capable of escaping vaccine-induced protection.

There are limited data describing the circulating genotypes in South Africa pre- and post-PCV13 introduction. We aimed to genetically characterize a comprehensive collection of invasive serotype 1 isolates, spanning 25 years, including whole-genome analyses of a subset of isolates to analyze temporal and/or vaccine pressure-associated genomic changes that may not be revealed by MLST.

(This work was presented in part at the 9th International Symposium on Pneumococci and Pneumococcal Diseases, Hyderabad, India, 9 to 13 March 2014 [23]).

## MATERIALS AND METHODS

### Historical isolates from 1979 to 1998.

Since 1979, the former South African Institute for Medical Research (whose functions were later absorbed by the National Health Laboratory Service in 2001) served as the national reference center for pneumococcal serotyping and monitoring of antimicrobial resistance (24–26). This was initiated following the discovery of the first multidrug-resistant pneumococcal isolate in 1977 in South Africa (27). Storage of isolates was not systematic; however, 23 randomly selected serotype 1 isolates believed to be representative of isolates collected at the time were available for characterization.

### IPD surveillance from 1999 to 2013.

Systematic national laboratory-based surveillance for IPD was initiated mid-1999 (28). Surveillance was enhanced in 2003 at select sentinel hospitals in all provinces, and additional clinical and demographic data were collected (2, 5). Approximately 200 laboratories throughout the country, which perform clinical microbiology diagnostic tests, submit reports of laboratory-confirmed IPD together with isolates to the reference laboratory at the National Institute for Communicable Diseases. IPD was defined as illness associated with the detection of S. pneumoniae from normally sterile site specimens. Quarterly regional laboratory audits identified previously unreported cases.

### Sampling strategy.

Serotype 1 data analyzed in this project were amalgamated from three different projects (see Table S1 in the supplemental material): for project 1, traditional MLST (i.e., PCR amplification and Sanger sequencing of 7 housekeeping genes) was performed on isolates with PCV serotypes collected prior to PCV introduction, namely, 2007 and 2011 (pre-PCV13). Isolates collected in 2012 and 2013 (early post-PCV13) were characterized by whole-genome sequencing. All viable isolates from children <5 years of age were selected for MLST. Due to the high numbers of isolates available for older individuals (≥5 years), half of all viable serotype isolates were selected for MLST. Where isolates failed to grow or yield an MLST result (due to nonamplification of some alleles or poor/incomplete sequence data), they were not replaced by selecting another isolate.

For project 2, whole-genome sequencing was performed on all available historical serotype 1 strains collected between 1989 and 1998 (*n* = 23), prior to the initiation of national laboratory-based surveillance for IPD. In addition, serotype 1 isolates collected over 15 years, from 1999 to 2013, through our national surveillance program for IPD were selected for whole-genome sequencing. The selection of isolates from each year was random, with a bias toward children (3:1 ratio of children to adults). For the selection process, children and adults were defined by ages of <15 years and ≥15 years, respectively.

For project 3, we sampled 300 invasive pneumococci per year over a period of 9 years (2005 to 2013) from isolates representing all serotypes, collected through our national surveillance. Isolate selection was random with respect to serotype and was stratified by age, namely, 150 isolates from children age 0 to 2 years, and 75 isolates each from children age 3 to 5 years and individuals who were ≥6 years. Data for serotype 1 isolates were extracted and included in this study.

### Phenotypic characterization.

Identification was confirmed by standard microbiological methods (29), and serotyping was performed by the Quellung reaction using serotype-specific antisera (Statens Serum Institut, Denmark). Antimicrobial susceptibility testing was performed by broth microdilution using Clinical and Laboratory Standards Institute guidelines and breakpoints, as previously described (2). Multidrug resistance was defined as nonsusceptibility to three or more of the following drug classes: penicillin, erythromycin, chloramphenicol, tetracycline, rifampin, or trimethoprim-sulfamethoxazole (co-trimoxazole).

### Molecular characterization.

Sampling of serotype 1 isolates for multilocus sequence typing (MLST) (*n* = 378) and whole-genome sequencing (*n* = 534) is summarized in Table S1 in the supplemental material. MLST was performed as previously described (30, 31). For genome sequencing, DNA was extracted from overnight broth cultures using the QIAamp DNA minikit (Qiagen, Inc., USA). Extracted DNA for 479/534 (90%) isolates was shipped to the Wellcome Trust Sanger Institute, United Kingdom, where paired-end whole-genome sequencing using index-tagged libraries was carried out using the Illumina HiSeq. The remaining 55 genomes were sequenced in South Africa; libraries were prepared using the Nextera XT DNA sample preparation kit (Illumina, USA), and sequencing was performed on an Illumina MiSeq.

### MLST analysis.

The eBURST version 3 algorithm was applied to generate population snapshots and determine founding genotypes and patterns of evolutionary descent between related sequence types (ST) (32). A clonal complex was defined as a cluster of related STs, whereby all STs were linked as single-locus variants (SLV) to another ST in the group. ST diversity was calculated using Simpson's diversity index (D). D ranges from 0 to 1, and values closer to 1 indicate higher diversity. STs were stratified by the age groups <5 years, 5 to 14 years, and >14 years, and PCV periods pre-PCV13 (1989 to 2011) versus early post-PCV13 (2012 to 2013).

### Genome assembly and annotation.

Internal automated pipelines developed by the Wellcome Trust Sanger Institute were used to assemble and annotate the genome. The assembly pipeline used Velvet, SSPACE, GapFiller, and SMALT (33–35) to produce a set of scaffold contigs, following which the annotation was run automatically on all assemblies with the pipeline using Prokka (36). STs were extracted using a script available at https://github.com/sanger-pathogens/mlst_check. The assembled reads were uploaded to PubMLST.org to identify the 53 ribosomal protein-encoding loci from the whole-genome data (37).

### Whole-genome phylogeny.

Phylogenetic relationships between isolates were assessed using whole-genome MLST (wgMLST) (31) and serotype 1 ST-227 (accession no. FQ312030) as an annotated reference genome. The selected BLASTN parameters used a cutoff of 70% identity over a 50% alignment, with a word size of 20 and core genome threshold of 90%. The distance matrix obtained for the genomes on the basis of core alleles was used to construct a phylogenetic tree using the neighbor-joining algorithm in SplitsTree version 4.13.1 (38).

### Recombination and resistance.

The sequence read data were mapped to the S. pneumoniae INV104B genome (serotype 1, ST-227, accession no. FQ312030) using SMALT (https://www.sanger.ac.uk/resources/software/smalt/) to produce a whole-genome alignment for all isolates. Genealogies Unbiased By recomBinations In Nucleotide Sequences (Gubbins) was used to identify regions within the genome alignment in which single nucleotide polymorphisms (SNPs) may have been introduced in a block by recombination (39). This iterative algorithm generated tab files showing the location of potential recombination, and a maximum-likelihood phylogeny was generated based on the putative point mutations outside these regions of high sequence diversity. The ratios of recombination events relative to point mutations (rho/theta), and of base substitutions imported through recombination to those occurring through point mutation (r/m), were calculated using the Gubbins output (19, 21). Antibiotic Resistance Gene-Annotation (ARG-ANNOT) was used to screen for the presence/absence of known antimicrobial resistance determinants (40).

### Statistical analyses.

Age-specific incidence rates for years 2003 to 2013 were calculated using population data available from Statistics South Africa (http://www.statssa.gov.za/). The χ^2^ test for trend was used to determine linear trends over time for incidence. Differences in proportions were assessed using the χ^2^ or Fisher's exact tests. Factors associated with rho/theta (ratio of the number of recombination events to point mutations) of serotype 1 overall and among the three ST-217 clades (as determined by whole-genome phylogeny) were assessed using linear regression. In addition, factors associated with ST-217 clades and antimicrobial susceptibility associated with the most commonly detected STs were assessed using multinomial regression. Multinomial regression allows modeling of outcome variables with more than two categories and relates the probability of being in category *j* to the probability of being in a baseline or reference category. A complete set of coefficients were estimated for each of the *j* levels (ST-217_C2_, ST-217_C3_, and other commonly detected STs) that were compared with the baseline category (ST-217_C1_), and the effect of each predictor in the model was measured as a relative risk ratio (RRR). ST-217_C1_ was chosen as the reference category, as it was the most represented cluster. Significance was assessed with two-sided *P* values of <0.05 for all models. Statistical analyses were implemented using Stata version 11 (StataCorp).

### Ethical considerations.

Ethical approval for national surveillance (which includes isolate characterization) was obtained through the human ethics research committee (medical) at the University of the Witwatersrand, Johannesburg, South Africa (protocol no. M140159).

### Nucleotide sequence accession numbers.

The newly deposited European Nucleotide Archive accession numbers are ERR1197531 to ERR1197628, ERR352058 to ERR352076, ERR387554 to ERR387648 (excluding ERR387589 and ERR387646), ERR568438, ERR568446, ERR568447, ERR568448, ERR568456, ERR568468, ERR568471, ERR568481, ERR568515, ERR568535, ERR568536, ERR568539, ERR568552, ERR568559, ERR568563, ERR568568, ERR568583, ERR568586, ERR568593, ERR568601, ERR568625, ERR568631, ERR568632, ERR568656, ERR568660, ERR568664, ERR568668, ERR568671, ERR568679, ERR568696, ERR568715, ERR568718, ERR568739, ERR568747, ERR568749, ERR570410, ERR570419, ERR570426, ERR570430, ERR570437, ERR570452, ERR570476, ERR586465, ERR586468, ERR600315, ERR600318, ERR600322, ERR600325, ERR600333, ERR600341, ERR600343, ERR600346, ERR600357, ERR600362, ERR600378, ERR600379, ERR600394, ERR600396, ERR600397, ERR600408, ERR600424, ERR600426, ERR600431, ERR600441, ERR600447, ERR600464, ERR600471, ERR600472, ERR600473, ERR600477, ERR600486, ERR600493, ERR600513, ERR600526, ERR600534, ERR600539, ERR600544, ERR600550, ERR600558, ERR600561, ERR600571, ERR600580, ERR600592, ERR600593, ERR600594, ERR600619, ERR632868, ERR632882, ERR632885, ERR632892, ERR632896, ERR632897, ERR632903, ERR632914, ERR632921, ERR632929, ERR632940, ERR632943, ERR632977, ERR632979, ERR632990, ERR632991, ERR633016, ERR633029, ERR633037, ERR633039, ERR633042, ERR633048, ERR633060, ERR633061, ERR633063, ERR633073, ERR633090, ERR633096, ERR633104, ERR633113, ERR646570, ERR646575, ERR646582, ERR646598, ERR646599, ERR701778, ERR701781, ERR701782, ERR701793, ERR701809, ERR701821, ERR701837, ERR701846, ERR701849, ERR701855, ERR701860, ERR708301, ERR714475, ERR714491, ERR714502, ERR714509, ERR714539, ERR714541, ERR714553, ERR714568, ERR714576, ERR714580, ERR714590, ERR714591, ERR714593, ERR714607, ERR714617, ERR714620, ERR714621, ERR714625, ERR714628, ERR714630, ERR714633, ERR714634, ERR714635, ERR714636, ERR714648, ERR714649, ERR714658, ERR714660, ERR714662, ERR714663, ERR714666, ERR714668, ERR714680, ERR714694, ERR714712, ERR730521, ERR730538, ERR730542, ERR730543, ERR730552, ERR730587, ERR730592, ERR730597, ERR730608, ERR730609, ERR730649, ERR730651, ERR730659, ERR730666, ERR730684, ERR730687, ERR730708, ERR730709, ERR730714, ERR730728, ERR730731, ERR730736, ERR730738, ERR730751, ERR730778, ERR730780, ERR730826, ERR730827, ERR730831, ERR730854, ERR730872, ERR730874, ERR730877, ERR730881, ERR730888, ERR730893, ERR730914, ERR730916, ERR730924, ERR736903, ERR736911, ERR736939, ERR773817. ERR773820, ERR773825, ERR773827, ERR773832, ERR773845, ERR773850, ERR773851, ERR773856, ERR773862, ERR773865, ERR773875, ERR773878, ERR773882, ERR773892, ERR773901, ERR773902, ERR773905, ERR773915, ERR773924, ERR773930, ERR773937, ERR773939, ERR773940, ERR773943, ERR773949, ERR773957, ERR773970, ERR773975, ERR773982, ERR773985, ERR773990, ERR773996, ERR774001, ERR774008, ERR774015, ERR774016, ERR774020, ERR774022, ERR774023, ERR774057, ERR774075, ERR774078, ERR774080, ERR774081, ERR774085, ERR774092, ERR774093, ERR774134, ERR774144, ERR774287, ERR774288, ERR774293, ERR774301, ERR774302, ERR774315, ERR774317, ERR774336, ERR774369, ERR774373, and ERR774376.

## RESULTS

### National surveillance from 1999 to 2013.

During this period, 53,647 cases of IPD were reported, of which 39,543 (74%) had viable isolates. From 2003 through 2009, the average incidences of serotype 1 disease were 1.1, 0.9, and 0.9 per 100,000 population in the <5 years, 5 to 14 years, and >14 years age groups, respectively. By 2013, the incidences declined to 0.19, 0.24, and 0.32 in the three age groups, respectively (*P* < 0.001). Serotype 1 was the most common IPD serotype in the pre- and post-PCV13 periods, accounting for 5,315 (13%) of the viable isolates (see Fig. S1A and B in the supplemental material). One percent (61/5,315) were nonsusceptible to penicillin, with an MIC_50_ of 0.12 μg/ml, compared to 37% (12,591/34,230) penicillin nonsusceptibility among non-serotype 1 isolates (MIC_50_, 0.25 μg/ml) (*P* < 0.001). Rates of serotype 1 nonsusceptibility to other antimicrobials were 12% (628/5,315), 6%, (324/5,315), 5% (252/5,315), 0.8% (41/5,315), 0.5% (28/5,315), and 0.3% (16/5,315) to co-trimoxazole, tetracycline, chloramphenicol, rifampin, erythromycin, and clindamycin, respectively. Multidrug resistance to any drug class was present in 4% (214/5,315) of serotype 1 isolates, whereas only 0.6% (32/5,315) were nonsusceptible to penicillin and two other drug classes. Epidemiological trends for serotype 1 disease from 2003 to 2013 have been described elsewhere (5).

### eBURST population structure.

**FIG 1:**
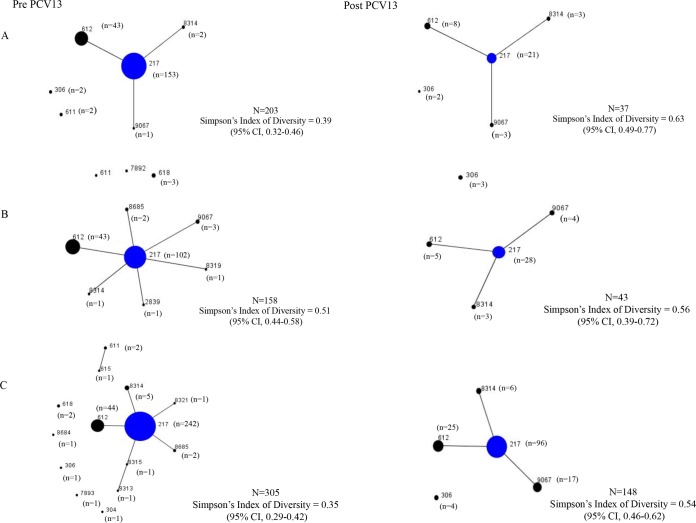
Population snapshot showing relationships between sequence types of invasive pneumococcal serotype 1 from children <5 years (A), 5 to 14 years (B), and >14 years (C), pre- and post-PCV13, South Africa, 1989 to 2013. Blue denotes founding genotype and circle size is indicative of number of isolates.

MLST data were available for 912 serotype 1 isolates; however, the population snapshots (Fig. 1A to C) include only those isolates for which patient age was known (*n* = 894). Sequence types, by age group and PCV period, are summarized in Table S2 in the supplemental material. Population snapshots were generated for each of the three age groups (<5 years, 5 to 14 years, and >14 years), and for pre- and early post-PCV13 periods. ST-217 accounted for 72% (642/894) of the isolates (excluding single-locus variants), and ST-217 clonal complex (including single-locus variants) accounted for 97% (866/894) of the isolates. Overall, ST diversity (D) was similar among isolates from all three age groups, namely, 0.43 (95% confidence interval [CI], 0.37 to 0.50), 0.53 (95% CI, 0.46 to 0.59), and 0.42 (95% CI, 0.36 to 0.47). ST diversity was relatively constant from 1999 to 2013 and trended toward increased diversity in 2012 and 2013 (see Fig. S2 in the supplemental material). Sequence type diversity (D), from the pre-PCV13 to post-PCV13 periods, increased from 0.39 to 0.63 (*P* = 0.002) in children <5 years and from 0.35 to 0.54 (*P* < 0.001) in individuals >14 years (Fig. 1A and C). Overall, the percentage of ST-217 isolates declined from 75% (153/203) to 57% (21/37) in children <5 years pre- and post-PCV13 introduction (*P* = 0.027). Similarly, in individuals >14 years, ST-217 declined from 79% (242/305) to 65% (96/148) (*P* = 0.001). ST-612 declined from 27% (43/158) to 12% (5/43) in the 5- to 14-year group after PCV13 introduction (*P* = 0.043). The newly reported ST-9067 increased proportionately from 0.6% (4/684) in the pre-PCV13 period to 11% (24/228) in the post-PCV13 period (*P* < 0.001). ST-9067 was detected only in younger individuals (0 to 14 years) prior to PCV-13 introduction, but was detected predominantly in older individuals (>14 years) post-PCV13 introduction (17/24 [71%]) (*P* = 0.016). In the 5- to 14-year age group, sequence type diversity remained unchanged (D, 0.51 versus 0.56, *P* = 0.616) before and after PCV13 introduction.

### Genomic population structure.

**FIG 2:**
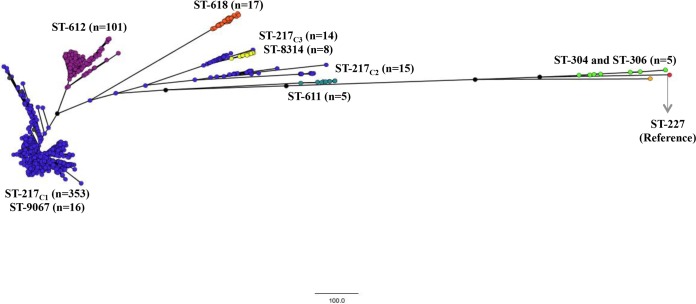
Maximum-likelihood phylogenetic tree, based on whole-genome single nucleotide polymorphisms outside recombination blocks, showing relationships between invasive serotype 1 isolates (*n* = 534) from South Africa, 1989 to 2013. Clusters are colored according to sequence type. ST-227 (accession no. FQ312030) was used as a reference.

Complete genomes were available for 534/912 (59%) isolates, with 428 and 106 genomes from the pre-PCV13 and early post-PCV13 periods, respectively. Using SNP analysis (Fig. 2) and wgMLST (see Fig. S3 in the supplemental material), the isolates clustered predominantly by sequence type. The largest clade (ST-217_C1_) comprised ST-217 (*n* = 353) and the newly reported SLV ST-9067 (*n* = 16). The ST-612 (SLV of ST-217) clade (*n* = 101) was the closest relative to ST-217_C1_, followed by triple-locus variant ST-618 (*n* = 17). Two additional ST-217 clades were identified (ST-217_C2_ [*n* = 15] and ST-217_C3_ [*n* = 14]), which clustered more distantly from ST-217_C1_. ST-217_C3_ isolates clustered with SLV ST-8314 (*n* = 8). Ribosomal MLST profiles differed among the three ST-217 clades: ribosomal ST 3462 (rST-3462) (*n* = 187/352 [53%]) and rST-3467 (*n* = 103/352 [29%]) were common among ST-217_C1_ isolates. The majority of ST-217_C2_ isolates (*n* = 13/15 [87%]) were rST-591, whereas ST-217_C3_ isolates were a mixture of four different ribosomal STs, with the most common being rST-4616 (5/14 [36%]). Multinomial logistic regression showed no association between ST-217 clades and gender, age group, or HIV status. ST-217_C3_ was more likely to cause disease in the post-PCV13 era than ST-217_C1_, but this was not statistically significant (RRR, 2.03; 95% CI, 0.617 to 6.709; *P* = 0.243).

### Recombination.

Genome characteristics, by sequence type, are summarized in Table 1. Using ST-217_C1_ as the reference group, the mean recombination ratios r/m and rho/theta did not vary significantly between sequence types, with the exception of ST-217_C3_, which had a mean r/m of 4.344 (95% CI, 2.951 to 5.739; *P* < 0.001) compared to ST-217_C1_ (mean r/m, 0.091), and ST-9067, which had a higher rho/theta (0.086) than that of ST-217_C1_ (0.013; 95% CI, 0.036 to 0.110, *P* < 0.001). Overall, rho/theta for ST-217 increased from 0.010 prior to PCV13 introduction to 0.039 post-PCV13 (*P* = 0.002). In the univariate analysis (Table 2), rho/theta among all serotype 1 isolates was higher post-PCV13 introduction (0.044 versus 0.010 [*P* < 0.001]) and in individuals >14 years (0.032 versus 0.008 [<5 years] [*P* = 0.002]). Serotype 1 isolates from HIV-positive individuals had a significantly lower rho/theta than that of isolates from HIV-negative individuals (0.001 versus 0.029, *P* = 0.018). In the multivariable model, only HIV status remained significantly associated with recombination (*P* = 0.019).

**TABLE 1:** Genome characteristics among sequence types (STs) of invasive serotype 1 pneumococcus, South Africa, 1989 to 2013

MLST	No. of isolates	Mean genome length (bp)	Mean (±SD) no. of SNPs outside recombination sites^*a*^	Mean (±SD) no. of recombination blocks^*b*^	Mean rho/theta^*c*^	*P* value^*d*^	Mean r/m^*e*^	*P* value^*d*^
ST-217 (All)	382	1,918,345	8 (±9)	0.24 (±1)	0.015		0.283	
ST-217_C1_	353	1,919,081	7 (±8)	0.16 (±0.7)	0.013	Ref	0.091	Ref
ST-217_C2_	15	1,903,232	13 (±11)	0.67 (±0.2)	0.035	0.253	0.934	0.220
ST-217_C3_	14	1,915,989	26 (±16)	1.64 (±4)	0.032	0.360	4.436	<0.001
ST-304	1	2,042,749	330	52	0.157	0.051	11.524	<0.001
ST-306	4	2,033,949	8 (±5)	0.25 (±0.43)	0.019	0.871	0.173	0.950
ST-611	5	1,934,828	16 (±22)	0.20 (±0.4)	0.009	0.911	0.076	0.990
ST-612^*f*^	101	1,915,591	8 (±8)	0.26 (±1)	0.012	0.866	0.610	0.078
ST-618	17	1,917,230	19 (±18)	0.06 (±0.2)	0.001	0.514	0.081	0.987
ST-8314^*f*^	8	1,900,792	7 (±4)	0.04 (±1)	0.038	0.357	0.625	0.567
ST-9067^*f*^	16	1,907,652	6 (±6)	0.56 (±1)	0.086	<0.001	0.813	0.279

a SNP, single nucleotide polymorphism.

b A recombination block was defined as ≥3 consecutive SNPs.

c rho/theta is the ratio of the number of recombination events to point mutations, a measure of the relative rates of recombination and point mutation (6, 11).

d ST-217_C1_ was the reference group for comparing rho/theta and r/m between sequence types.

e r/m is the ratio of base substitutions predicted to have been imported through recombination to those occurring through point mutation.

f Single-locus variant of ST-217.

**TABLE 2:** Univariate and multivariable analyses of the ratio of recombination events to point mutations (rho/theta) among invasive serotype 1 pneumococci (all sequence types), South Africa, 1989 to 2013

Variable	Serotype 1 (all sequence types)		Univariate analysis		Multivariable analysis	
	No. of genomes	Mean rho/theta^*a*^	Regression coefficient	*P* value	Regression coefficient	*P* value
PCV period^*b*^						
Pre-PCV13	428	0.010				
Post-PCV13	106	0.044	0.035	<0.001	0.022	0.120
Gender						
Female	226	0.017				
Male	273	0.017	0.000	0.971		NA^*c*^
Age group (yr)						
<5	188	0.008				
5–14	144	0.010	0.002	0.780	0.014	0.348
>14	184	0.032	0.024	0.002	0.024	0.109
HIV status						
Negative	52	0.029				
Positive	67	0.001	−0.028	0.018	−0.031	0.019

a rho/theta is the ratio of the number of recombination events to point mutations, a measure of the relative rates of recombination and point mutation (6, 11).

b Pre-PCV13 was defined as 1989 to 2011; early post-PCV13 was defined as 2012 to 2013.

c NA, not applicable.

### Antimicrobial resistance.

The majority of ST-217_C3_ isolates were nonsusceptible to co-trimoxazole (13/14 [93%]), and 29% (4/14) were nonsusceptible to chloramphenicol and tetracycline (Table 3). For ST-8314 isolates, 90% (18/20), 85% (17/20), 70% (14/20), and 15% (3/20) were nonsusceptible to co-trimoxazole, chloramphenicol, tetracycline, and penicillin, respectively. In the multinomial model, compared to ST-217_C1_, ST-217_C2_, ST-217_C3_, and ST-8314, isolates were significantly associated with increased nonsusceptibility to co-trimoxazole, chloramphenicol, and tetracycline (*P* < 0.001). In addition, ST-8314 was associated with increased nonsusceptibility to penicillin (*P* < 0.001). *tet*(M) and chloramphenicol acetyltransferase (*cat*_pC194_) genes were confirmed in ST-217_C2_, ST-217_C3_, and ST-8314 isolates with phenotypic nonsusceptibility. The conjugative transposon Tn*916* was confirmed to flank *tet*(M) in these isolates.

**TABLE 3:** Multinomial logistic regression analysis of antimicrobial nonsusceptibility among ST-217, ST-612, ST-8314, and ST-9067 of invasive serotype 1 pneumococcus, South Africa, 1989 to 2013

Antimicrobial agent^*a*^	Resistance data by MLST (*n*)^*b*^											
	ST-217_C1_ (353)		ST-217_C2_ (15)		ST-217_C3_ (14)		ST-612 (168)		ST-8314 (20)		ST-9067 (28)	
	No. (%) resistant	RRR (95% CI), *P* value	No. (%) resistant	RRR (95% CI), *P* value	No. (%) resistant	RRR (95% CI), *P* value	No. (%) resistant	RRR (95% CI), *P* value	No. (%) resistant	RRR (95% CI), *P* value	No. (%) resistant	RRR (95% CI), *P* value
PEN	2 (0.6)	Reference	0 (0)		0 (0)		3 (2)	3.192 (0.528–19), 0.206	3 (15)	31 (5–198), <0.001	0 (0)	
CHL	1 (0.3)	Reference	14 (93)	4,928 (293–82,917), <0.001	4 (29)	141 (14–1,376), <0.001	1 (0.6)	2 (0.1321–34), 0.599	17 (85)	1,995 (197–20,194), <0.001	0 (0)	
TET	1 (0.3)	Reference	15 (100)	4,923 (293–82,766), <0.001	4 (29)	141 (14–1,376), <0.001	2 (1)	4 (0.382–47), 0.240	14 (70)	821 (93–7,290), <0.001	0 (0)	
ERY	0 (0)	Reference	0 (0)		0 (0)		1 (0.6)		1 (5)		0 (0)	
CLI	0 (0)	Reference	0 (0)		0 (0)		0 (0)		0 (0)		0 (0)	
RIF	2 (0.6)	Reference	0 (0)		0 (0)		1 (0.6)	1.051 (0.095–11), 0.967	0 (0)		0 (0)	
TMP-SMX	14 (4)	Reference	14 (93)	339 (42–2,757), <0.001	13 (93)	315 (38–2,575), <0.001	6 (4)	0.897 (0.338–2.377), 0.827	18 (90)	218 (46–1,032), <0.001	0 (0)	

a PEN, penicillin; CHL, chloramphenicol; TET, tetracycline; ERY, erythromycin; CLI, clindamycin; RIF, rifampin; TMP-SMX, trimethoprim-sulfamethoxazole (co-trimoxazole).

b RRR, relative risk ratio; 95% CI, 95% confidence interval.

## DISCUSSION

Serotype 1 remains the most common serotype causing IPD in South Africa; however, reductions in incidence were noted post-PCV13 introduction. ST-217 remained predominant throughout the period of analysis, although increases in ST diversity were observed post-PCV13 introduction. A decline in ST-217 and ST-612 was observed, whereas newly reported ST-9067 increased in proportion in the sampled population. Although largely susceptible to all antimicrobials tested, there were sublineages of ST-217 associated with increased nonsusceptibility to chloramphenicol, tetracycline, and co-trimoxazole. ST-8314 was also associated with increased nonsusceptibility to penicillin.

ST-217 accounted for the large majority of serotype 1 isolates. Although this lineage is common to Africa and Israel (16, 41), early isolates were identified in northern Europe and, due to its global dissemination, it was designated Sweden^1^-27 by the Pneumococcal Molecular Epidemiology Network (PMEN) (42, 43). During the late 1980s, Sweden experienced an increase in pneumococcal bacteremia due to serotypes 1 and 14. Although MLST identified predominantly the European/North American clone (ST-306) among the Swedish serotype 1 isolates, ST-217 was identified in one isolate. ST-217 has also been reported in the United States, albeit rarely (3, 15).

Our data set has serotype 1 ST-217 isolates dating back to 1991, and the fact that this clone remained predominant throughout the 25-year period indicates a measure of genetic stability among serotype 1 isolates in South Africa. However, sequence type diversity increased in young children (<5 years) and older individuals (>14 years) following PCV13 introduction. Most noteworthy was the overall decline in the percentage of ST-217 isolates and the apparent expansion of newly reported ST-9067 within the sampled data set. ST-9067 had a significantly higher recombination ratio than that of ST-217_C1_, which may explain its possible expansion following PCV13 introduction; however, the impetus for this is unknown, given that it is fully susceptible to all tested antimicrobials. In the United States, the increase in serotype 19A post-PCV introduction was thought to be driven, at least in part, by the expansion of antibiotic-resistant clonal complex 320 (CC320) (44). Genomic surveillance of pre- and post-PCV invasive pneumococci of all serotypes from adults in the Netherlands showed a temporary decline in genetic diversity following PCV7 use (45).

Phylogenetic analyses revealed three distinct clades within the ST-217 lineage, indicating a level of resolution beyond that of the traditional 7-locus MLST. This subdivision of the ST-217 clone was not evident in a recent analysis of serotype 1 isolates (which included 58 genomes from South Africa), presumably because of the smaller sample size and shorter time frame (5 years) in which the South African isolates were collected for that study (17). Whole-genome phylogeny revealed the unusual clustering of ST-9067 and ST-8314 with ST-217_C1_ and ST-217_C3_ isolates, respectively. It also showed that ST-217_C1_ was more closely related to SLV ST-612 (an older more established lineage detectable in South Africa since 2000) than ST-217_C2_ or ST-217_C3_. ST-611 and ST-618 isolates, neither of which belong to the ST-217 clonal complex, were also more closely related to ST-217_C1_ than ST-217_C2_ and ST-217_C3_. Nevertheless, ST-618 shares four MLST alleles with ST-217, indicating some degree of relatedness and potentially justifying the clustering with ST-217_C1_. There were several additional ST-217 SLVs that emerged in the same year that PCV13 was introduced (2011); however, these sequence types were not sustained and disappeared in 2012. We were not able to assess their relationships relative to the other sequence types at the whole-genome level, as we did not have those data.

Since pneumococcal diversification is predominantly due to genetic exchange caused by interaction with other pneumococci and closely related streptococcal species in the nasopharynx, a short duration of carriage is thought to limit this exchange and thus stabilize the genome. The recombination ratio gives an indication of how diversity is arising in the population. Croucher et al. (19) showed a high degree of recombination in a single multidrug-resistant pneumococcal lineage (PMEN1, Spain^23F^-1), in which 88% diversity was introduced by recombination. Recombination ratios for isolates representing multidrug-resistant globally disseminated clones (19, 22, 46, 47) were significantly higher (ranging from 7.2 to 34.1) than those for our ST-217_C1_ and ST-217_C2_ clades (0.09 and 0.93, respectively). The recombination ratio for ST-217_C3_ was significantly higher than that for ST-217_C1_ at 4.4 and more comparable to that of PMEN lineages. The recently published global serotype 1 genomic study reported similarly low recombination ratios, ranging from 0.03 (for the South African and Mozambique isolates) to 0.14 (for the West African isolates) (17).

The short duration of carriage of serotype 1 is possibly responsible for the low levels of antibiotic resistance (6). Genomic analyses of >3,000 pneumococcal carriage isolates showed that recombination coincided with genes associated with antibiotic resistance (21). In our study, crude analysis of the recombination hotspots showed increased recombination at the *folP* locus (together with *folC* and *folE*, both of which are involved in folate biosynthesis) in the co-trimoxazole-resistant ST-217_C2_ and ST-217_C3_ clades (data not shown). Co-trimoxazole is widely used in South Africa as a prophylactic in HIV patients, and S. pneumoniae has been shown to have high rates of nonsusceptibility to this drug (48). Nonetheless, serotype 1 was predominantly susceptible to co-trimoxazole and, more importantly, penicillin, which remains the drug of choice for the treatment of pneumococcal pneumonia. The multidrug-resistant ST-217_C2_ and ST-217_C3_ clades accounted for a small proportion of isolates overall (<10%) and may either proliferate under the selective pressure of co-trimoxazole use or may decline altogether as serotype 1 IPD declines through continued PCV13 use. If ST-8314 were to expand, this would be of greater concern because of its association with penicillin resistance. Almost half (8/17) of the penicillin-susceptible ST-8314 isolates had MICs of 0.06 μg/ml (data not shown), indicating a trend toward nonsusceptibility. Multidrug resistance can be acquired through transposons, which may carry one or several resistance determinants. Their integration causes changes to the host genome and, in this study, these changes were reflected through the identification of ST-217 subclades. Tetracycline and/or macrolide resistance has been shown to be associated with the Tn*916*/Tn*1545* family of conjugative transposons, and, in particular, Tn*916*, which carries the *tet*(M) resistance determinant (49).

An interesting observation was the significantly lower recombination ratio (rho/theta) observed in serotype 1 isolates from HIV-positive individuals compared to that of HIV-negative individuals. This might seem surprising given the relatively high pneumococcal colonization rate observed among HIV-infected adults compared with those who are HIV uninfected (50), which should increase the reservoir of genes available for genetic exchange. Nevertheless, reduced selective pressure from an immunosuppressed host may limit the need for genetic exchange.

This analysis includes approximately 20% of serotype 1 isolates collected during this period, with sampling bias for some years, and nonsystematic collection of historical isolates prior to 1999. Thus, our data set may not accurately reflect temporal or vaccine-associated changes in serotype 1. Only 2 years of post-PCV13 data were included; hence, potentially consequential vaccine-associated genotypic changes are probably unlikely to have occurred at this stage. Nevertheless, this study highlights the predominance of a successful globally disseminated serotype 1 clone, with increased sequence diversity in the sampled population, following PCV13 introduction. Antimicrobial-nonsusceptible subclades provide the potential to become more widespread and merit the continued monitoring of serotype 1 genotypes following PCV13 introduction.

## Supplementary Material

Supplemental material
