# Supplementary material for: Phylogenetic Analysis of Invasive Serotype 1 Pneumococcus in South Africa, 1989 to 2013
Source: J Clin Microbiol. 2016 Apr 25;54(5):1326–34. doi: 10.1128/JCM.00055-16 (PMC4844715; doi:10.1128/JCM.00055-16)
Supplement: Supplemental material [file supp_54_5_1326__index.html]

Supplemental material 

# Phylogenetic Analysis of Invasive Serotype 1 Pneumococcus in South Africa, 1989 to 2013

## Supplemental material

- Supplemental file 1 -

  Tables S1 (Isolate selection for genetic characterization of invasive serotype 1 pneumococcus, South Africa, 1989 to 2013) and S2 (Sequence type distribution among invasive serotype 1 pneumococcus isolates, South Africa, 1989 to 2013, by age and PCV13 period) and Fig. S1A (Common serotypes causing invasive pneumococcal disease in South Africa, 1999 to 2011, by age group), S1B (Common serotypes causing invasive pneumococcal disease in South Africa, 2011 to 2013, by age group), S2 (Simpson’s index of diversity for invasive serotype 1 pneumococcus sequence types among individuals of all ages in South Africa, 1999 to 2013, by year), and S3 (Neighbor-joining tree showing relationships between serotype 1 isolates from South Africa, 1989 to 2013, using whole-genome MLST)

  PDF, 374K
