# Supplementary material for: Phylogenetic Analysis of Invasive Serotype 1 Pneumococcus in South Africa, 1989 to 2013
Source: J Clin Microbiol. 2016 Apr 25;54(5):1326–34. doi: 10.1128/JCM.00055-16 (PMC4844715; doi:10.1128/JCM.00055-16)
Supplement: Supplemental material [file JCM.00055-16_zjm005164944so1.pdf]

Supplementary Table 1. Isolate selection for genetic characterization of invasive serotype 1 pneumococcus, South Africa, 1989-2013 (N=912)

Year	No. of reported serotype 1 cases[a]	Whole genome sequencing (MLST)[b]				
		Age				
		<5 years	5-14 years	>14 years	Unknown	Total
1989	-				2	2
1991	-	3			2	5
1995	-			1	7	8
1996	-				7	7
1999	171	5	5	3		13
2000	295	6	3	2		11
2001	265	6	5	3		14
2002	223	3	5	4		12
2003	567	4	7	4		15
2004	554	4	4	4		12
2005	486	16	14	15		45
2006	374	12	12	17		41
2007	330	17 (26)	17 (75)	21 (13)		55 (114)
2008	390	26	12	12		50
2009	476	20	9	14		43
2010	371	16	20	13		49
2011	352	25 (14)	10 (104)	11 (22)		46 (140)
2012	300	17 (11)	8 (22)	11 (86)		36 (119)
2013	161	8	13	49		70
**Total**	**5315**	**188 (52)**	**144 (57)**	**184 (269)**	**18**	**534 (378)**

[a] Laboratory-based surveillance was initiated mid-1999. Only cases for which a viable isolate was available are recorded in the table. Sampling prior to 1999 was based on availability and viability of isolates collected for special studies.

[b] Traditional MLST was done on isolates shown in parentheses () and thus whole genome sequencing data are not available for these isolates.

Supplementary Table 2. Sequence type distribution among invasive serotype 1 pneumococcus, South Africa, 1989-2013, by age and PCV13 period (N=894[a])

Sequence type (clonal complex)	No. isolates per age group and by PCV period					
	1989-2011 (pre-PCV13)			2012-2013 (early post-PCV13)		
	<5 years	5-14 years	>14 years	<5 years	5-14	>14 years
217 (217)	153	102	242	21	28	96
612 (217)	43	43	44	8	5	25
2839 (217)		1				
8313 (217)			1			
8314 (217)	2	1	5	3	3	6
8315 (217)			1			
8319 (217)		1				
8321 (217)			1			
8685 (217)		2	2			
9067 (217)	1	3		3	4	17
304			1			
306	2		1	2	3	4
611	2	1	2			
615			1			
618		3	2			
7892		1				
7893			1			
8684			1			
**TOTAL**	**203**	**158**	**305**	**37**	**43**	**148**

[a] Age data were not recorded for 18 cases preceding 1999, prior to the initiation of national, laboratory-based surveillance

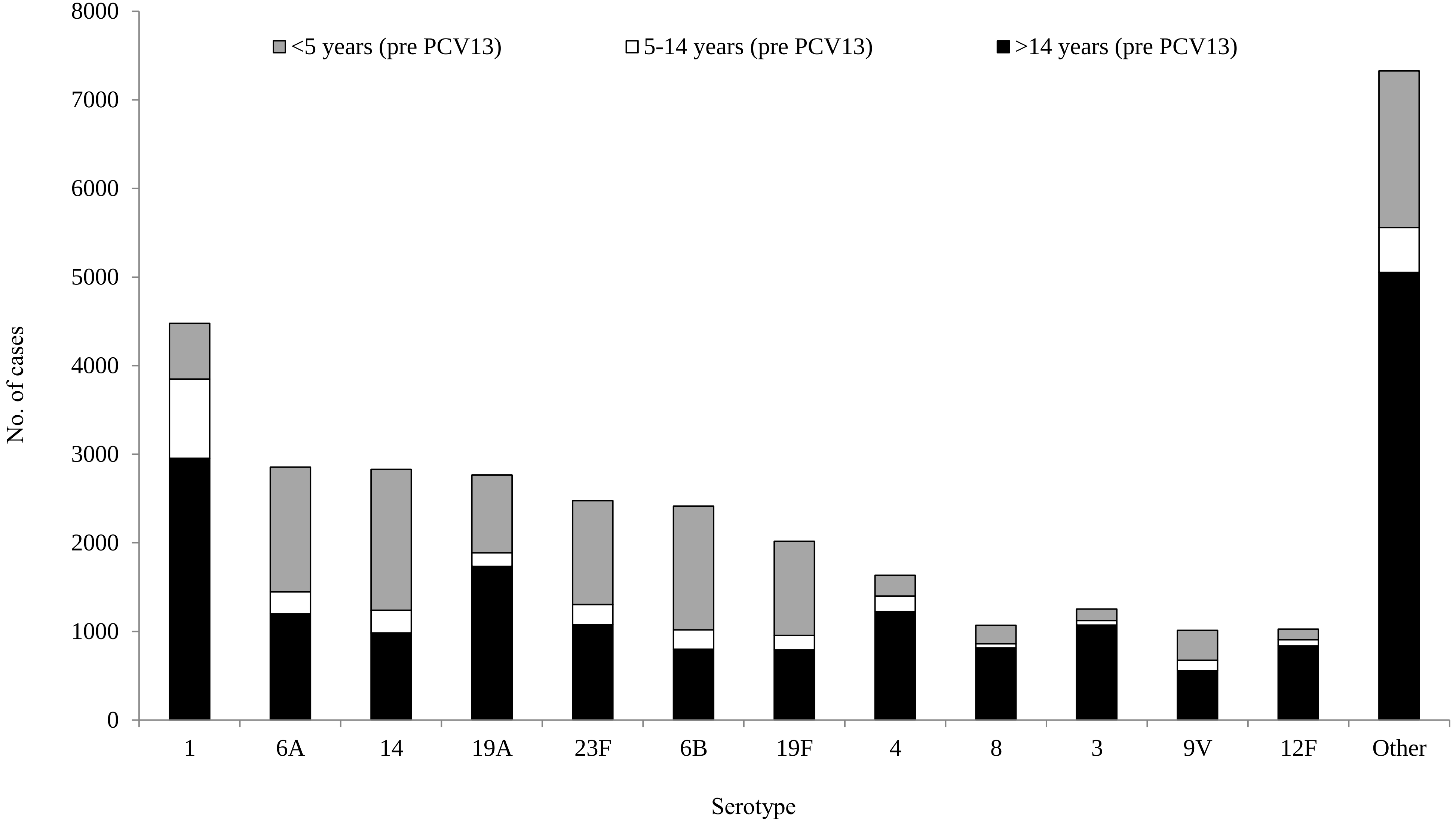

Supplementary Figure 1A. Common serotypes (in order of decreasing prevalence) causing invasive pneumococcal disease in South Africa, 1999-2011 (pre-PCV13), by age group (N=33,158). 'Other' indicates all other serotypes not individually listed. Nontypeable isolates and isolates for cases where age data are missing have been excluded.

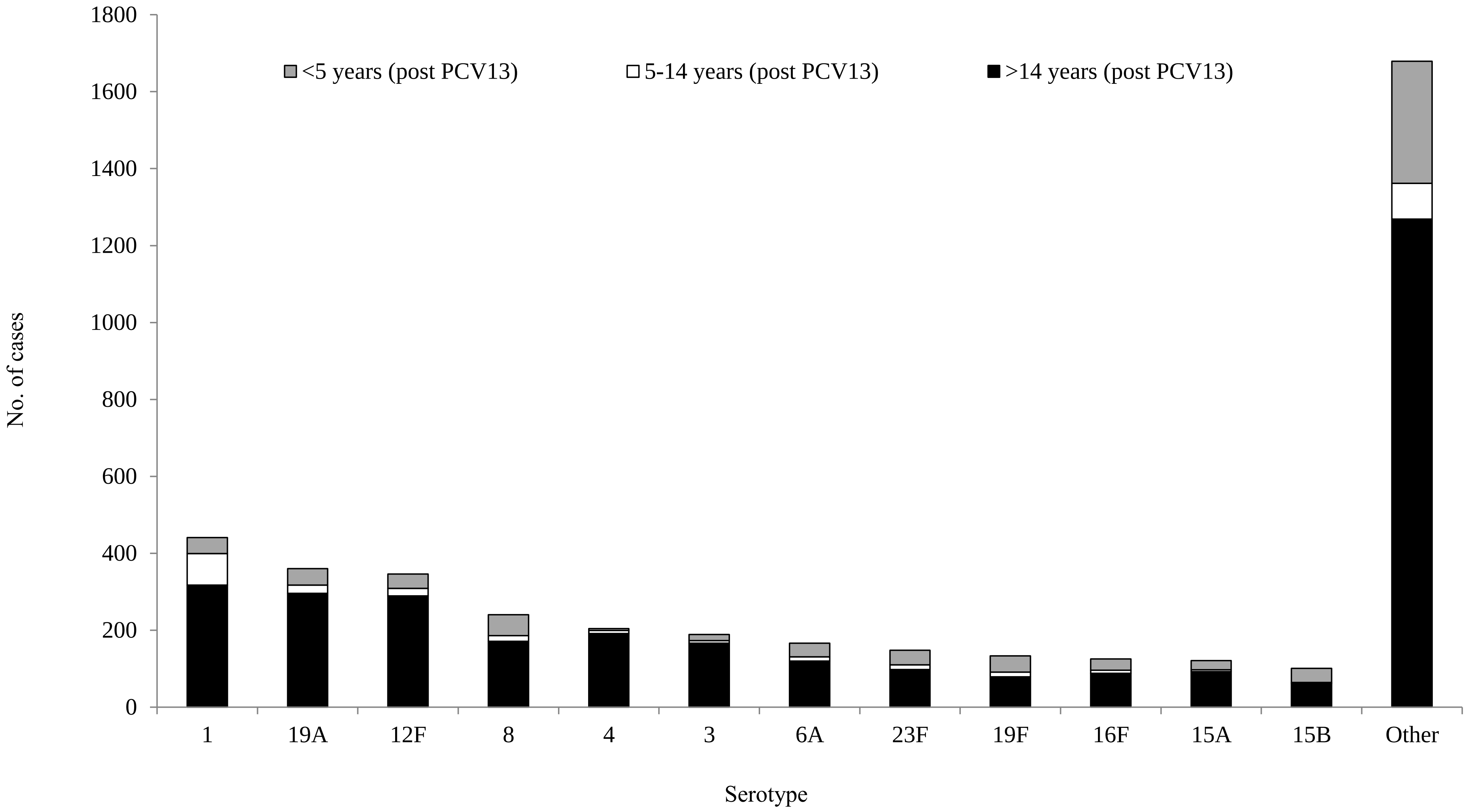

Supplementary Figure 1B. Common serotypes (in order of decreasing prevalence) causing invasive pneumococcal disease in South Africa, 2011-2013 (early post-PCV13), by age group (N=3938). ‘Other’ indicates all other serotypes not individually listed. Nontypeable isolates and isolates for cases where age data are missing have been excluded.

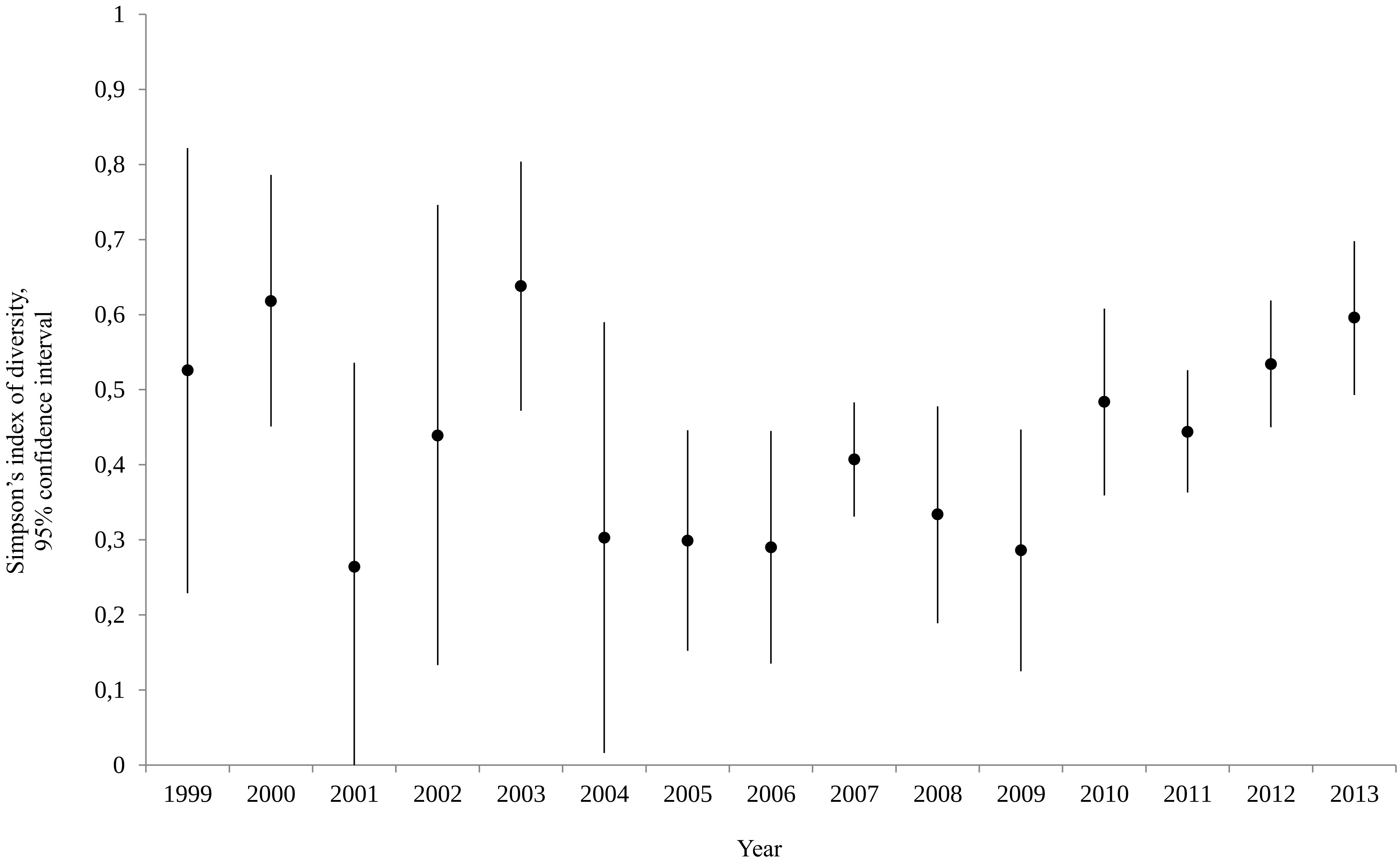

Supplementary Figure 2. Simpson's index of diversity for invasive serotype 1 pneumococcus sequence types among individuals of all ages, by year, South Africa, 1999-2013

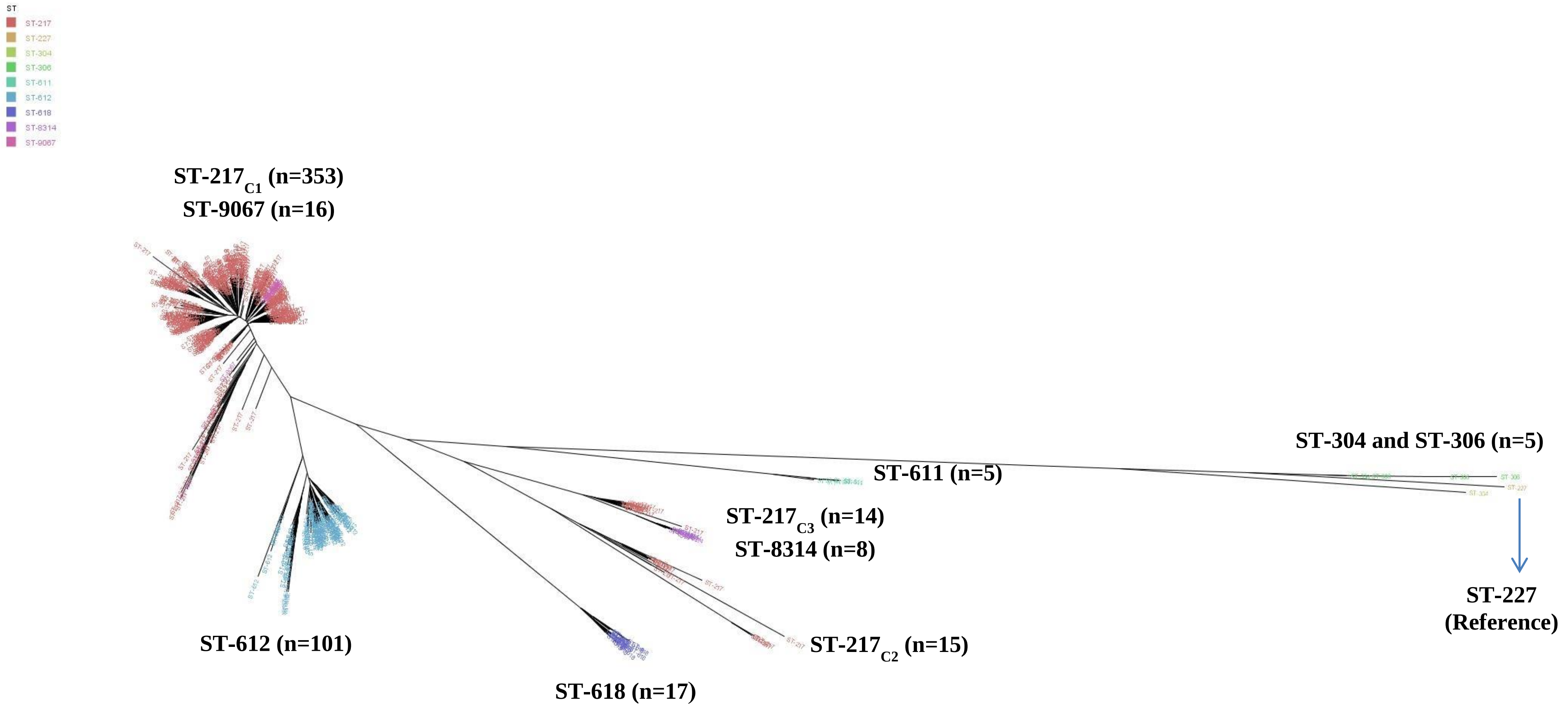

Supplementary Figure 3. Neighbour joining tree showing relationships between serotype 1 isolates (N=534) from South Africa, 1989-2013, using whole genome MLST. Clusters are coloured according to sequence type (ST). ST-227 (Accession no. FQ312030) was used as a reference.
